# Magnetic Activated-ATP@Fe_3_O_4_ Nanocomposite as an Efficient Fenton-Like Heterogeneous Catalyst for Degradation of Ethidium Bromide

**DOI:** 10.1038/s41598-017-06398-3

**Published:** 2017-07-20

**Authors:** Shuwen Han, Hemin Yu, Tingting Yang, Shengsen Wang, Xiaozhi Wang

**Affiliations:** 1 College of Environmental Science and Engineering, Yangzhou University, Jiangsu, 225127 China; 2 Jiangsu Collaborative Innovation Center for Solid Organic Waste Resource Utilization, Nanjing, 210095 China

## Abstract

Magnetic attapulgite-Fe_3_O_4_ nanocomposites (ATP-Fe_3_O_4_) were prepared by coprecipitation of Fe_3_O_4_ on ATP. The composites were characterized by scanning electron microscopey, X-ray diffractometry, Brunauer-Emmett-Teller analysis, X-ray photoelectron spectroscopy, energy dispersive spectrometer and transmission electron microscopy. Surface characterization showed that Fe_3_O_4_ particles with an average size of approximately 15 nm were successfully embedded in matrix of ATP. The capacity of the Fe_3_O_4_-activated ATP (A-ATP@Fe_3_O_4_) composites for catalytic degradation of ethidium bromide (EtBr, 80 mg/L) at different pH values, hydrogen peroxide (H_2_O_2_) concentrations, temperatures, and catalyst dosages was investigated. EtBr degradation kinetics studies indicated that the pseudo-first-order kinetic constant was 2.445 min^−1^ at *T* = 323 K and pH 2.0 with 30 mM H_2_O_2_, and 1.5 g/L of A-ATP@Fe_3_O_4_. Moreover, a regeneration study suggested that A-ATP@Fe_3_O_4_ maintained over 80% of its maximal EtBr degradation ability after five successive cycles. The effects of the iron concentrations and free radical scavengers on EtBr degradation were studied to reveal possible catalytic mechanisms of the A-ATP@Fe_3_O_4_ nanocomposites. Electron Paramagnetic Resonance revealed both hydroxyl (∙OH) and superoxide anion (∙O_2_^−^) radicals were involved in EtBr degradation. Radical scavenging experiment suggested EtBr degradation was mainly ascribed to ∙OH radicals, which was generated by reaction between Fe^2+^ and H_2_O_2_ on the surface of A-ATP@Fe_3_O_4_.

## Introduction

Ethidium bromide (EtBr) is widely employed for rapid visualization of nucleic acids in electrophoretic gels and is commonly used in the life sciences^1^. However, due to its strong toxicity and powerful mutagenicity^2^, EtBr is detrimental to human health and could cause hepatocellular carcinoma and some infectious diseases. To remediate EtBr contamination, sorption^3^ and degradation techniques^4, 5^ are commonly adopted for EtBr removal. Although sorption has been proven for its reasonable EtBr removal efficiency, the sorbed EtBr requires further treatment.

Advanced oxidation technologies for wastewater treatment have attracted attention due to the generation of strongly oxidizing hydroxyl radical (∙OH)^6–9^. Hydroxyl radicals, a very active and efficient non-selective oxidant, are capable of degrading organic pollutants into such harmless endproducts as carbon dioxide (CO_2_) and H_2_O^10^. Heterogeneous Fenton technology has gained popularity recently, especially that employing nano-sized magnetite (Fe_3_O_4_) as a catalyst^11, 12^. Nano-sized Fe_3_O_4_ has a similar catalytic activity with horseradish peroxidase, which can effectively decompose hydrogen peroxide (H_2_O_2_) into ∙OH^13^. Fe_3_O_4_ nanoparticles can initiate Fenton reaction through the following mechanisms, eg. (i) Fe^2+^ can act as an electron donor to initiate the Fenton reaction according to the classical Haber–Weiss mechanism, (ii) Fe^2+^ and Fe^3+^ can be easily accommodated on the octahedral site in the magnetite structure, where Fe^2+^ can be oxidized and thereafter reduced back to the same structure^14^, and (iii) the presence of multiple oxidation states of iron (Fe^2+^ and Fe^3+^) in magnetite enhances decomposition of hydrogen peroxide^15^. However, the Fe_3_O_4_ nanoparticles with high surface energies and intrinsic magnetic inter-action tend to aggregate that would reduce surface/volume ratio and dispersion stability in aqueous solution, and thus compromise the catalytic activity^16^. Therefore, to improve degradation catalytic ability, Fe_3_O_4_ nanoparticles are commonly supported by clay minerals such as bentonite^17^, carbon nano-material^18, 19^, and fly ash^20^, etc. The supported nanoparticles are characterized with enhanced activities.

Attapulgite (ATP) is a crystalline hydrated magnesium silicate mineral. It has an unusual layer-chain crystal structure with a large number of microporous channels and a relatively high surface area, which assign it remarkable adsorption ability. ATP has good sorptive removal capacity for metallic^21, 22^ and organic contaminants^23, 24^ in aqueous solutions. However, ATP could not degrade organic contaminants. Besides, colloidal ATPs are hard to be separated from aqueous solutions^25^. Instead, Fe_3_O_4_ nanoparticles can not only degrade organic contamiants by generating free radicals, but are paramagnetic which facilitate easy separation, from aqueous solution^26^. Thus, ATP supported Fe_3_O_4_ nanoparticles is not only capable of degradation of organic contaminants, but easy to be seperated from solutions. Further, ATP has a good cation exchange capacity and thus can attract Fe^2+^ and Fe^3+^ to its surfaces. The sorbed Fe^2+^ and Fe^3+^ can react with -OH to generate Fe(OH)_2_ and Fe(OH)_3_, respectively. Although Fe_3_O_4_-ATP nanomaterials have been reported by a few researchers for removal of hazardous metal ions from water^27, 28^, their use as a catalyst in the heterogeneous Fenton reaction has rarely been reported. In this study, the catalytic properties of Fe_3_O_4_-ATP nanomaterials for degradation of EtBr and the relevant mechanisms were investigated.

Since Fe_3_O_4_-ATP nanoparticles simultaneously exhibit adsorptive and catalytic, we anticipate that Fe_3_O_4_-ATP nanoparticles are a powerful candidate for catalytic activation of H_2_O_2_. This paper presents our research on the catalytic properties of superparamagnetic nanoscaled Fe_3_O_4_-ATP composite, which was used to promote Fenton oxidation of EtBr by H_2_O_2_. Thereby, Fe_3_O_4_-ATP nanoparticles were prepared and their physical and chemical characteristics were determined. The applicability of this composite in heterogeneous Fenton reaction was evaluated in view of the effect of the main variables (pH, temperature and H_2_O_2_ concentration, and catalyst dosage), reaction kinetics, and material stability, as well as the degradation mechanism.

## Results and Discussion

### Characterization of catalysts

**Figure 1:**
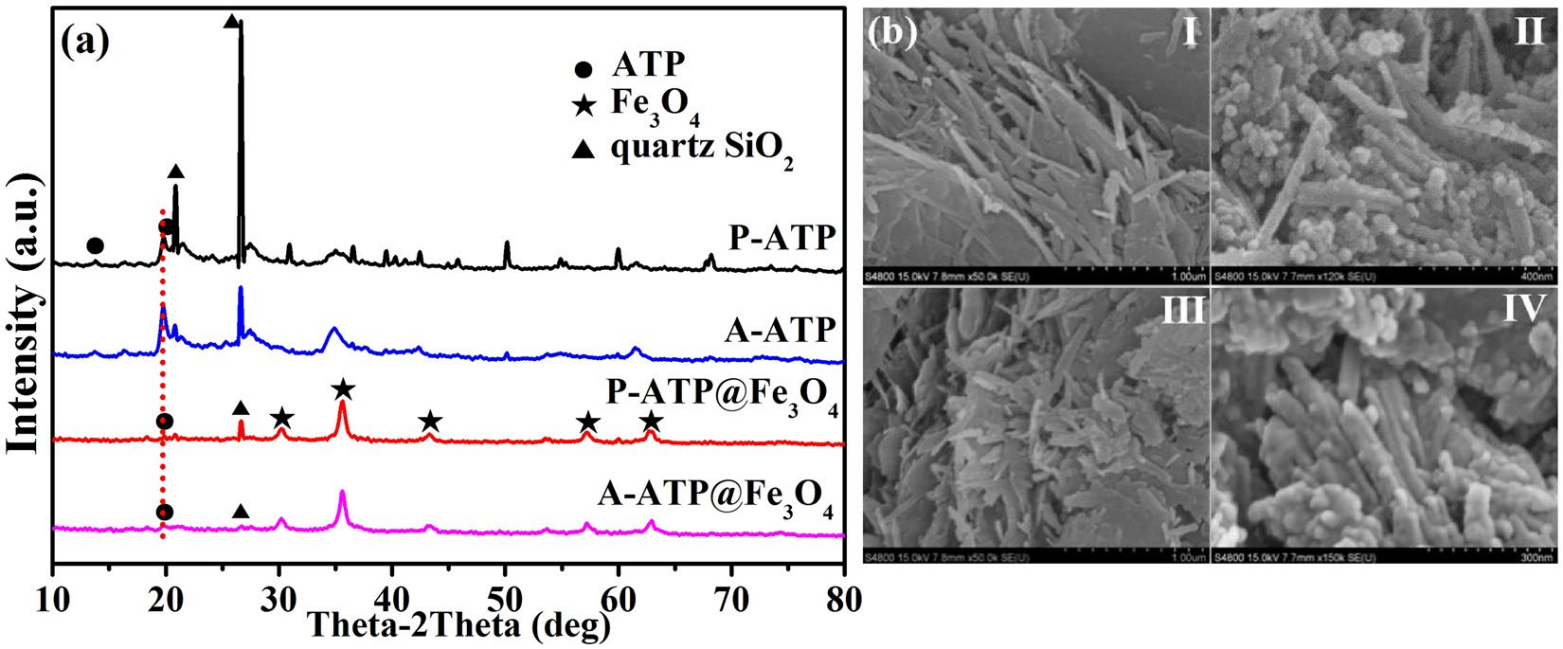
(**a**) XRD patterns and corresponding diffraction peaks for P-ATP@Fe_3_O_4_ and A-ATP@Fe_3_O_4_. The original P-ATP and A-ATP serves as control. (**b**) SEM images of (I) P-ATP, (II) P-ATP@Fe_3_O_4_, (III) A-ATP, (IV) A-ATP@Fe_3_O_4_.

The powder X-ray diffraction (XRD) characteristic peaks of purified ATP (P-ATP) and activated ATP (A-ATP) (Fig. 1(a)) at 2*θ* = 13.7° and 19.8° are consistent with (200) and (040) planes of ATP^29^. The peaks at 2*θ* = 20.9° and 26.6° correspond to quartz (SiO_2_) in P-ATP and A-ATP^28^, but the intensity of diffraction peaks of quartz SiO_2_ was weakened in A-ATP. The XRD patterns of Fe_3_O_4_ in the ATP@Fe_3_O_4_ composites all exhibited cubic spinel structure (JCPDS 65–3170), as evidenced by the weak diffraction peaks from the (220), (311), (400), (511) and (440) planes at 30.1°, 35.5°, 43.1°, 56.9° and 62.6°, respectively^30, 31^. The intensities of the ATP peak at 2*θ* = 19.8° and the quartz peaks at 2*θ* = 20.9° and 26.6° were weaker for the Fe_3_O_4_-purified ATP (P-ATP@Fe_3_O_4_) and Fe_3_O_4_-activated ATP (A-ATP@Fe_3_O_4_), indicating that Fe_3_O_4_ nano-particles were embedded in the ATP. But the characteristic reflections for ATP were observed in all of ATP@Fe_3_O_4_ composites, suggesting that modification process did not destroy the characteristic structure of ATP.

The average crystallite size (*D*) of Fe_3_O_4_ particles on the surface of A-ATP was 12.8 nm, as estimated with Scherrer equation^30^. The scanning electron microscopy (SEM) images (Fig. 1(b,IV)) show that Fe_3_O_4_ particles were distributed regularly on the rod-like structure of ATP. The SEM images indicate that the average size of the Fe_3_O_4_ particles was about 15 nm, which matches well with XRD results.

**Figure 2:**
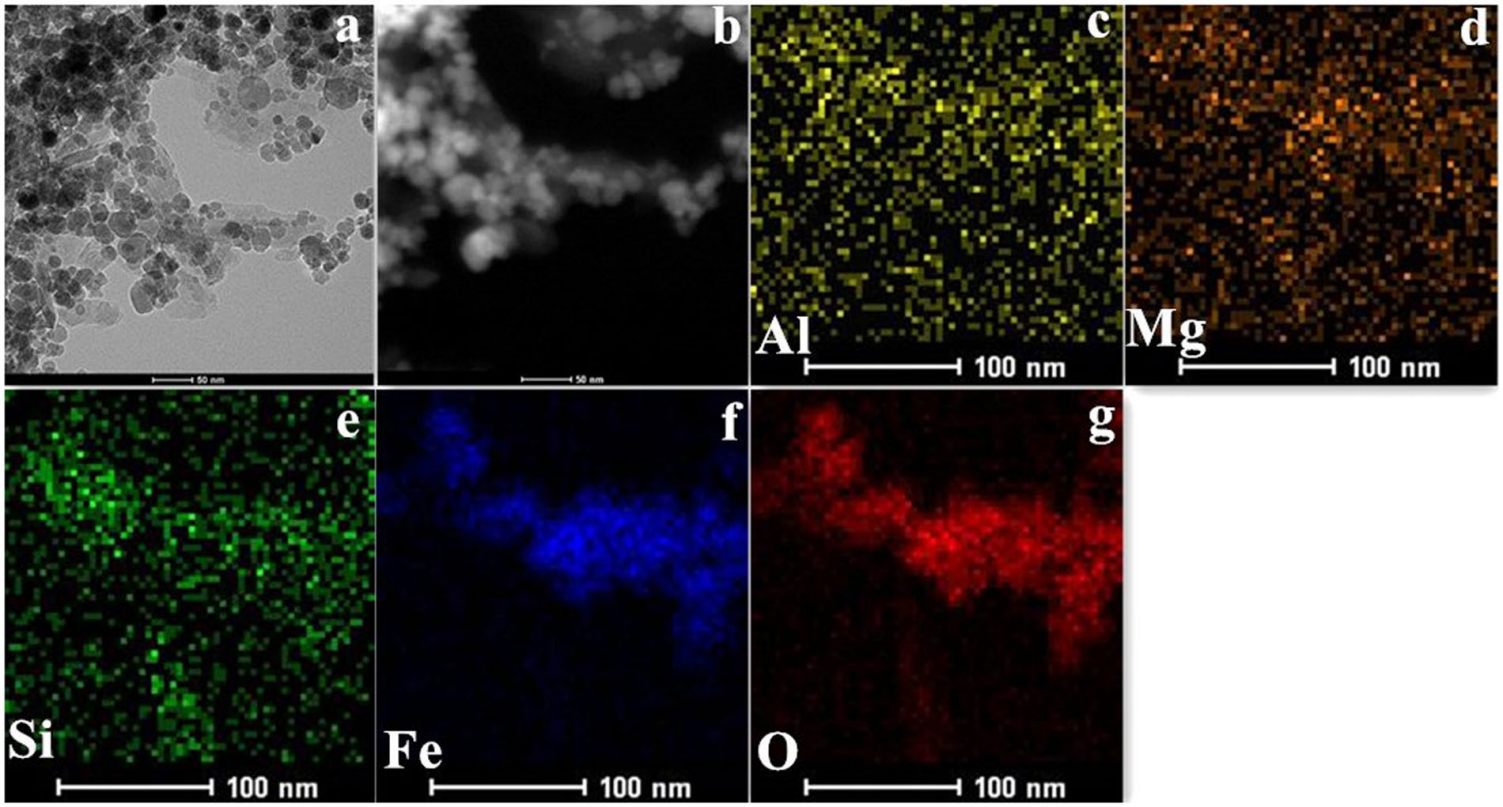
TEM images of (**a**) A-ATP@Fe_3_O_4_; (**b**) HAADF-STEM images of A-ATP@Fe_3_O_4_; (**c**–**g**) the corresponding EDX elemental mapping of aluminum, magnesium, silicon, iron, oxygen.

Transmission electron microscopy (TEM) images show that the materials exhibited rod like structure with highly uniform rotundness and void sizes (Fig. 2(a)), in agreement with the results of SEM analysis. The particles size ranged from 10–15 nm, which is consistent with the XRD result. Elemental mapping images (Fig. 2(c–g)) of A-ATP@Fe_3_O_4_ confirmed that the presence of Fe and O atoms in nanocomposites with a content of above 30% and 50% (Table 1). All of these results demonstrated that the Fe_3_O_4_ nanoparticles were loaded on the ATP.

**Table 1:** The atomic content of corresponding elemental of A-ATP@Fe_3_O_4_ (%).

Material	Al	Mg	Si	Fe	O
A-ATP@Fe_3_O_4_	3.62	1.78	12.15	29.81	52.00

The nitrogen adsorption/desorption isotherms of Fe_3_O_4_, P-ATP@Fe_3_O_4_, and A-ATP@Fe_3_O_4_ (Supplementary information Fig. S1(a)) all illustrated a typical type IV pattern with a bend of volume adsorption of nitrogen at a *P/P*_*0*_ value of approximately 0.5 with a H_3_-type hysteresis loop. This pattern indicates the presence of mesoporous structure. In addition, the presence of mesoporous structure is also confirmed by the Barrett–Joyner–Halenda (BJH) corresponding pore size distribution curve (see Supplementary Fig. S1(b)). Furthermore, the typical type IV pattern with a H_3_-type hysteresis loop also illustrated that the nanomaterials comprised of aggregates (loose assemblages) of platelike (rod-like) particles forming slitlike pores^32^. The Brunauer–Emmett–Teller (BET) surface area, pore size, and pore volume of A-ATP@Fe_3_O_4_ were 125.2745 m^2^/g, 11.80 nm, and 0.3695 cm^3^/g, respectively (Table 2). In particular, the specific surface area was about 1.5 times larger than that of other reported catalysts^33^.

**Table 2:** Summary of physicochemical properties of P-ATP, A-ATP, Fe_3_O_4_, P-ATP@Fe_3_O_4_, and A-ATP@Fe_3_O_4_.

sample	S_*BET*_ (m^2^/g)	pore size (nm)	pore volume (cm^3^/g)	*M*_*s*_ (emu/g)	30 min of adsorption removal rate
P-ATP	173.72	8.21	0.36	—	95%
A-ATP	190.25	7.51	0.36	—	97%
Fe_3_O_4_	85.18	14.5	0.31	62.61	15%
P-ATP@ Fe_3_O_4_	100.17	12.4	0.31	44.78	51%
A-ATP@ Fe_3_O_4_	125.27	11.8	0.37	41.78	64%

**Figure 3:**
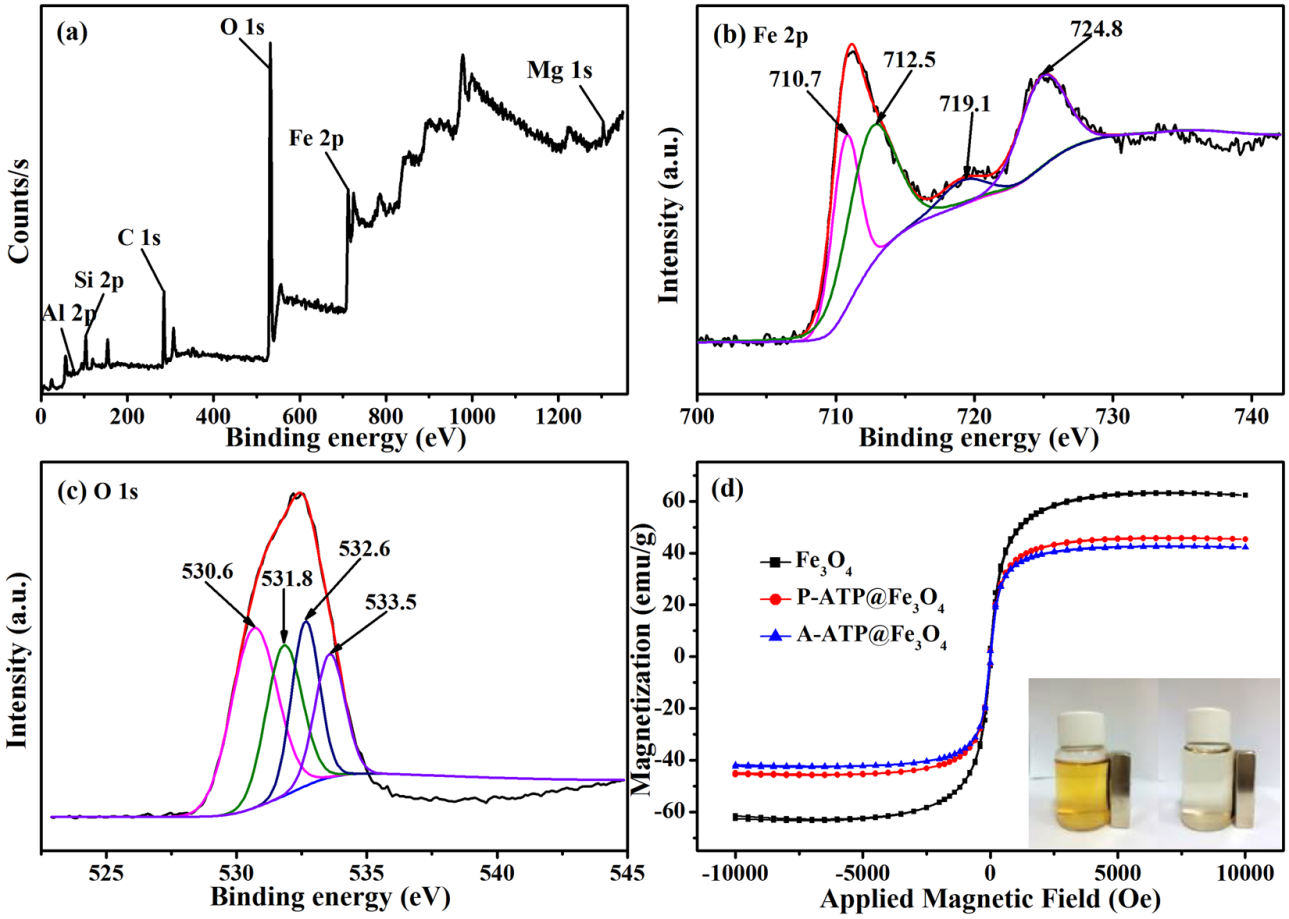
(**a**) XPS spectra of A-ATP@Fe_3_O_4_, high-resolution XPS spectra of (**b**) Fe2p, (**c**)O1s. (**d**) Magnetization curves of P-ATP@Fe_3_O_4_ and A-ATP@Fe_3_O_4_ nanocomposites and Fe_3_O_4_ serves as control. The inset pattern is a photograph of the magnetic separation, which shows that the nanoparticles can be separated easily with a magnet.

The surface elemental composition of A-ATP@Fe_3_O_4_ was obtained (Fig. 3(a)) by X-ray photoelectron spectroscopy (XPS) analysis, and the binding energy (BE) for Mg 1s, Fe 2p, O 1s, Si 2p and Al 2p were 1304.08, 712.08, 532.08, 103.08 and 87.08 eV, respectively.

Four peaks were observed in the Fe 2p spectrum of A-ATP@Fe_3_O_4_ (Fig. 3(b)). The Fe 2p_3/2_ peak with a BE of 710.7 eV was indicative of Fe^3+^ octahedral species^34^. The BE of 724.8 eV for Fe 2p_1/2_ indicated the presence of octahedrally coordinated Fe^2+^ ^34^, or ferric iron oxides (Fe_3_O_4_)^35^. The relative lower BE peak at 712.5 eV is attributed to Fe^3+^, with a corresponding Fe^3+^ satellite at 719.1 eV, which furthermore confirmed that both Fe^2+^ and Fe^3+^ were present in the nanocomposites.

Figure 3(c) showed the O 1s XPS spectrum of A-ATP@Fe_3_O_4_. The spectrum can be fitted to four peaks with BEs of 530.6, 531.8, 532.6, and 533.5 eV. The peak at 530.6 eV resulted from the lattice oxygen in Fe_3_O_4_^36^. The two peaks at 531.8 and 532.6 eV were attributed to the monodentate oxygen atoms (H–O) and monodentate and bidentate oxygen species (Si–O–Si), respectively^36, 37^. The intensity ratios of the monodentate to bidentate oxygen atoms showed that surface of A-ATP@Fe_3_O_4_ was primarily bidentate. The remaining peak at 533.5 eV could be assigned to the chemically equivalent oxygen in the bidentate bond (O–C=O)^38^. For A-ATP@Fe_3_O_4_ catalyst, chemisorbed oxygen is the most active oxygen species which plays an important role in the oxidation reaction.

The Fourier transform infrared (FT-IR) spectra of the P-ATP, A-ATP, P-ATP@Fe_3_O_4_ and A-ATP@Fe_3_O_4_ samples are shown in Supplementary Fig. S2. In the spectrum of P-ATP and A-ATP, the absorbance bands at 3357 cm^−1^ and 3617 cm^−1^ were ascribed to the O–H stretching vibration of structural water and other water molecules in ATP^39^. The characteristic bands of stretching vibration of Si–O–Si for P-ATP and A-ATP was observed around 1033 cm^−1^, as well as the bending vibration of H–O–H located at 1652 cm^−1^. These three typical adsorption bands were also observed for P-ATP@Fe_3_O_4_ and A-ATP@Fe_3_O_4_, but the absorption peaks were all weaker than those of the samples not loaded with Fe_3_O_4_, which implies that crystallization was essentially completely^40^. In addition, the peak at 582 cm^−1^ was owing to Fe–O bond for Fe_3_O_4_ was observed in both P-ATP@Fe_3_O_4_ and A-ATP@Fe_3_O_4_^41^. All the results confirmed that the Fe_3_O_4_ nanoparticles were loaded on the ATP.

The hysteresis loops of Fe_3_O_4_, P-ATP@Fe_3_O_4_, and A-ATP@Fe_3_O_4_ were investigated to observe their magnetization property. Figure 3(d) clearly showed that the three curves all exhibit almost zero remanence and coercivity, indicating that three types of nanoparticles were superparamagnetic^42^. The saturation magnetization (*M*_*s*_) values were found to be 62.61, 44.78 and 41.78 emu/g for Fe_3_O_4_, P-ATP@Fe_3_O_4_, and A-ATP@Fe_3_O_4_, respectively. However, the “dilution effects” of ATP result in lower, *M*_*s*_ values of the P-ATP@Fe_3_O_4_ and A-ATP@Fe_3_O_4_ particles relative to Fe_3_O_4_ particles^43^. Superparamagnetism is shown in Fig. 3(d) (pattern in inset) and demonstrated that the synthesized particles could be easily separated from solution by applying an external magnetic field. This phenomenon suggests an especially important advantage of our catalyst because it could be used for recycling.

### Catalytic activity of ATP-Fe_3_O_4_ composite

**Figure 4:**
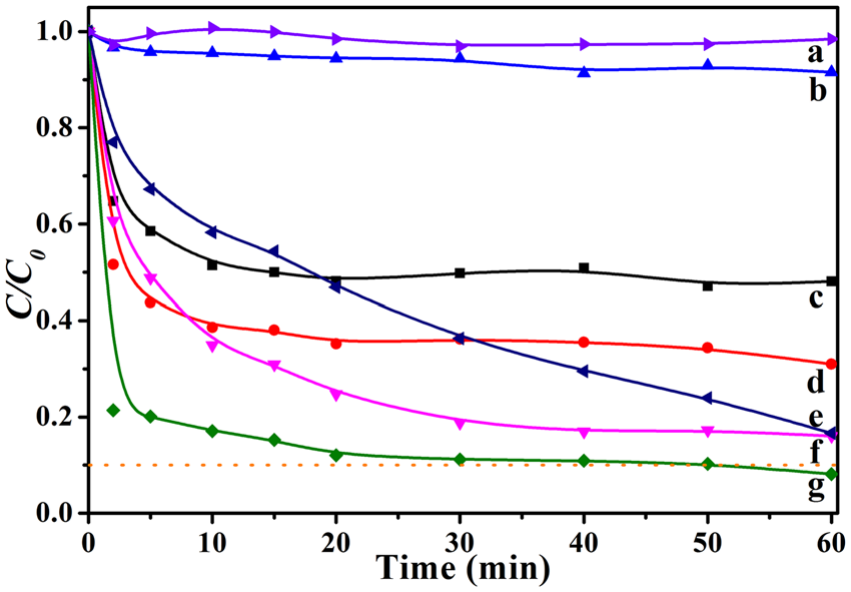
Removal of EtBr under different conditions: (a) 30 mM H_2_O_2_. (b) 1.5 g/L Fe_3_O_4_ without H_2_O_2_. (c) 1.5 g/L P-ATP@Fe_3_O_4_ without H_2_O_2_. (d) 1.5 g/L A-ATP@Fe_3_O_4_ without H_2_O_2_. (e) 1.5 g/L Fe_3_O_4_ with 30 mM H_2_O_2_. (f) 1.5 g/L P-ATP@Fe_3_O_4_ with 30 mM H_2_O_2_. (g) 1.5 g/L A-ATP@Fe_3_O_4_ with 30 mM H_2_O_2_. Other reaction conditions were initial EtBr concentration 80 mg/L, pH 2.0, and *T* = 323 K, *C*_*0*_ and *C* are initial EtBr concentration after 30 min adsorption and its concentration at any time during the reaction, respectively.

To compare the efficiency of EtBr removal by various processes, the control experiments with Fe_3_O_4_ only were investigated at pH 2.0 with initial EtBr concentration of 80 mg/L. After 30 min of dark adsorption, P-ATP@Fe_3_O_4_ and A-ATP@Fe_3_O_4_ showed better EtBr removal rates (51% and 64% respectively) than Fe_3_O_4_ alone (15%) (Table 1). The EtBr removal was ascribed mainly to the surface adsorption by ATP and Fe_3_O_4_ minerals. The enhanced EtBr sorption by A-ATP was due to the increased surface area. The degradation reaction results (Fig. 4) showed that H_2_O_2_ yielded only negligible removal of EtBr within 60 min. In the presence of H_2_O_2_, the degradation rate of EtBr using the Fe_3_O_4_-ATP composite was notably higher than that for Fe_3_O_4_, implying that the catalytic activity was enhanced by the introduction of ATP. After a 60 min heterogeneous Fenton reaction, Fe_3_O_4_-ATP composites exhibited a removal rate of 94%. In addition, the catalytic activity of A-ATP@Fe_3_O_4_ composite was higher than those of P-ATP@Fe_3_O_4_ or Fe_3_O_4_. The degradation rates within 20 min reached 90% for A-ATP@Fe_3_O_4_ composite but only 50% for Fe_3_O_4_, suggesting a synergetic effect in the A-ATP@Fe_3_O_4_ composite. The enhanced degradation rate of A-ATP@Fe_3_O_4_ nanoparticles may be ascribed to increased surface area of Fe_3_O_4_ nanoparticles which were well dispersed on the surface of A-ATP. The relative rates of mass transfer to reactive sites and chemical reaction at reactive sites would thus be enhanced.

**Figure 5:**
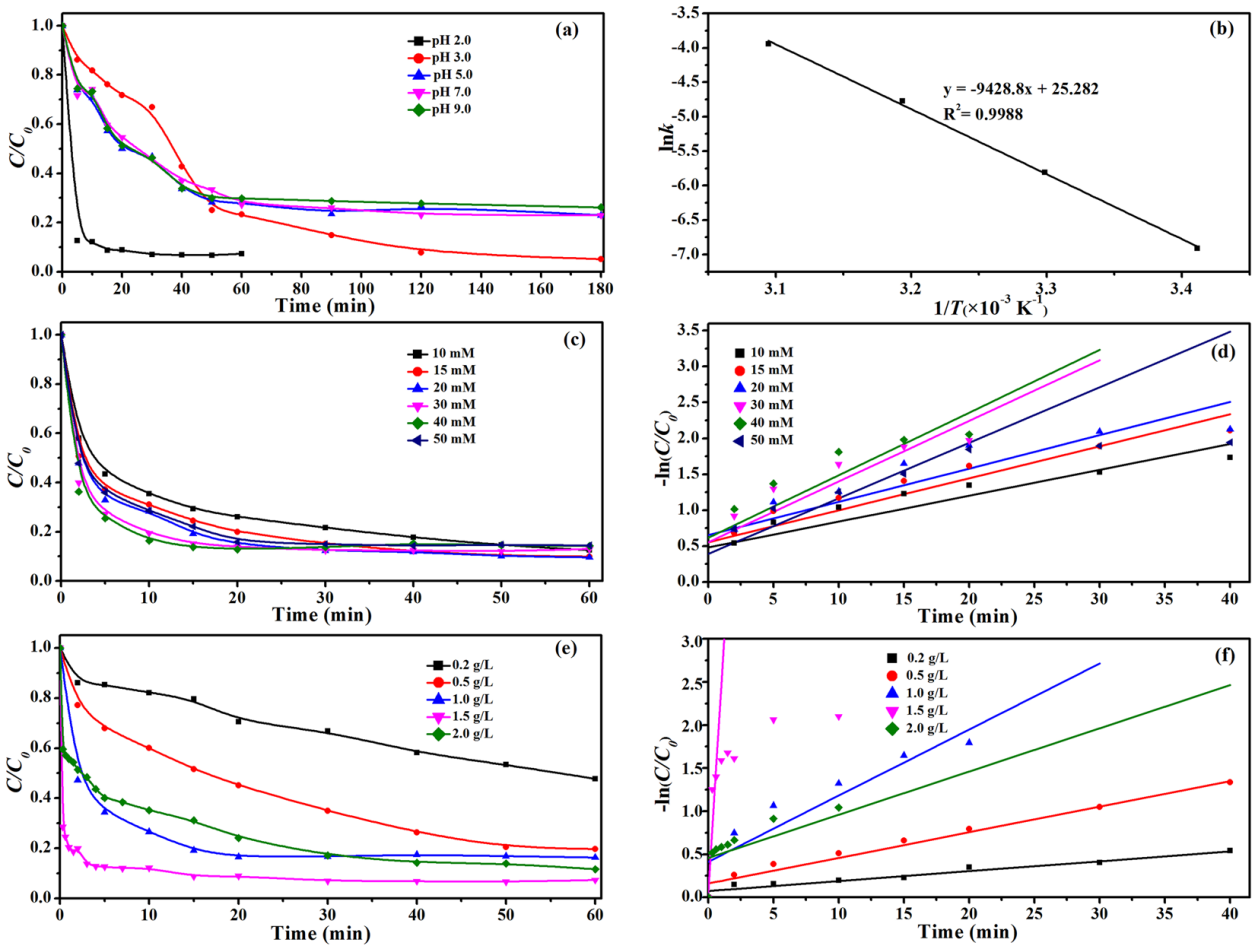
Factorial effects of heterogeneous Fenton reaction on EtBr (80 mg/L) degradation by A-ATP@Fe_3_O_4_: (**a**) initial pH value, and (**b**) Arrhenius plot based on the effect of temperature. (**c**) H_2_O_2_ dosage, (**e**) A-ATP@Fe_3_O_4_ composite addition, (**d**) and (**f**) Pseudo-first-order kinetics corresponding to (**c**) and (**e**) (where the slope of the plot of −ln(*C/C*_*0*_) versus reaction time is the apparent degradation rate constant *k*). Except for the investigated parameter, other parameters were fixed: catalysts load 1.5 g/L, initial H_2_O_2_ concentration 30 mmol/L and the temperature of the system 323 K of (**a**), catalysts load 1 g/L, pH 2.0, initial H_2_O_2_ concentration 15 mmol/L of (**b**). And catalysts load 1 g/L, pH 2.0, the temperature of the system 323 K of (**c**) and (**e**), pH 2.0, initial H_2_O_2_ concentration 30 mmol/L and the temperature of the system 323 K of (**d**) and (**f**).

The pH effects on EtBr degradation by A-ATP@Fe_3_O_4_ catalyst was determined (Fig. 5 (a)). About 78% of EtBr was removed after 180 min of reaction at pH 5. EtBr degradation increased as pH decreased, suggesting that the production of ∙OH on the surface of A-ATP@Fe_3_O_4_ was limited at higher pHs. Although EtBr removal rates decreased between pH 3 and 9, the nanocomposites still exhibited good EtBr degradation capacity. This implied that A-ATP@Fe_3_O_4_ exhibited strong catalytic activity in a wide range of pH values. Under acidic, neutral, and alkaline conditions, the EtBr degradation was content with a pseudo first order reaction in kinetics, which might be expressed as ln(*C*_*t*_ */C*_*0*_) = *kt* + *m*, where *m* is a constant, *k* is the apparent rate constant (min^−1^), *C*_*0*_ is the residual concentration of EtBr (mmol/L) after 30 min absorption and *C*_*t*_ is EtBr concentration at different sampling times. The highest *k* values of 2.445 min^−1^ for EtBr degradation was observed at pH 2.0, and thus pH 2.0 was selected for subsequent experiments.

The kinetics of EtBr degradation was also investigated at different temperatures (293, 303, 313, and 323 K). The activation energy (*E*_*a*_) of the reaction was evaluated by plotting ln*k* against *1/T* (Fig. 5(b)) according to the Arrhenius equation. The activation energy was determined to be 78.39 kJ/mol for A-ATP@Fe_3_O_4_. This *E*_*a*_ value falls within a reasonable range from the literature of 60 to 250 kJ/mol^44^. Dependence on the temperature in a heterogeneous Fenton-like reaction was previously reported through a carbon-Fe structured catalyst for the degradation of orange II with an activation energy of 56.1 kJ/mol (in a similar temperature range)^45^. Thus, the heterogeneous catalytic reaction of A-ATP@Fe_3_O_4_/H_2_O_2_ is geared to a general chemical reaction. This result indicated that the heterogeneous Fenton-like reaction of A-ATP@Fe_3_O_4_/H_2_O_2_ does not require a very high energy. The effects of H_2_O_2_ concentration and A-ATP@Fe_3_O_4_ dosage at pH 2.0 and *T* = 323 K on the catalytic activity of A-ATP@Fe_3_O_4_. As shown in Fig. 5(d), the apparent rate constant *k* increased from 0.0361 to 0.844 min^−1^ as the increase of H_2_O_2_ dosage from 10 to 30 mmol/L, being about 2 folds of nanoscaled Fe_3_O_4_/CeO_2_ composite reported by Xu *et al*.^46^ According to the classical Haber–Weiss mechanism^47^, Fe^2+^ induces hydrogen peroxide to generate hydroxyl radicals (∙OH), and the ∙OH can then react with Fe^3+^ to regenerate Fe^2+^ that can circularly produce ∙OH radicals in the Fenton reaction. However, *k* value declined to 0.446 min^−1^ at a higher H_2_O_2_ concentrations of 40 mmol/L. It is possibly related to the scavenging effect of ∙OH radicals when excessive H_2_O_2_ inhibits the production of ∙OH radicals^48^. To shorten reaction time, a higher concentration of H_2_O_2_ (30 mmol/L) was applied for EtBr removal. In this case, it is necessary to investigate the loading of A-ATP@Fe_3_O_4_ (Fig. 5(f)). In our study, as the amount of A-ATP@Fe_3_O_4_ increased from 0.2 to 2.0 g/L, the rate constant *k* of EtBr degradation first increased and then decreased sharply. The increased removal rate may be due to the production of more reactive oxidants resulting from more active sites at higher rates of A-ATP@Fe_3_O_4_. The severe depression of EtBr removal is possibly ascribed to the scavenging of ∙OH radicals by excess Fe^2+^ ^49^. To conclude, better removal of EtBr with a shorter reaction time can be achieved under the following conditions: the 1.5 g/L of A-ATP@Fe_3_O_4_, pH = 2.0, *T* = 323 K, and 30 mmol/L H_2_O_2_ (standard reaction condition).

### Effect of Fe ion release

**Figure 6:**
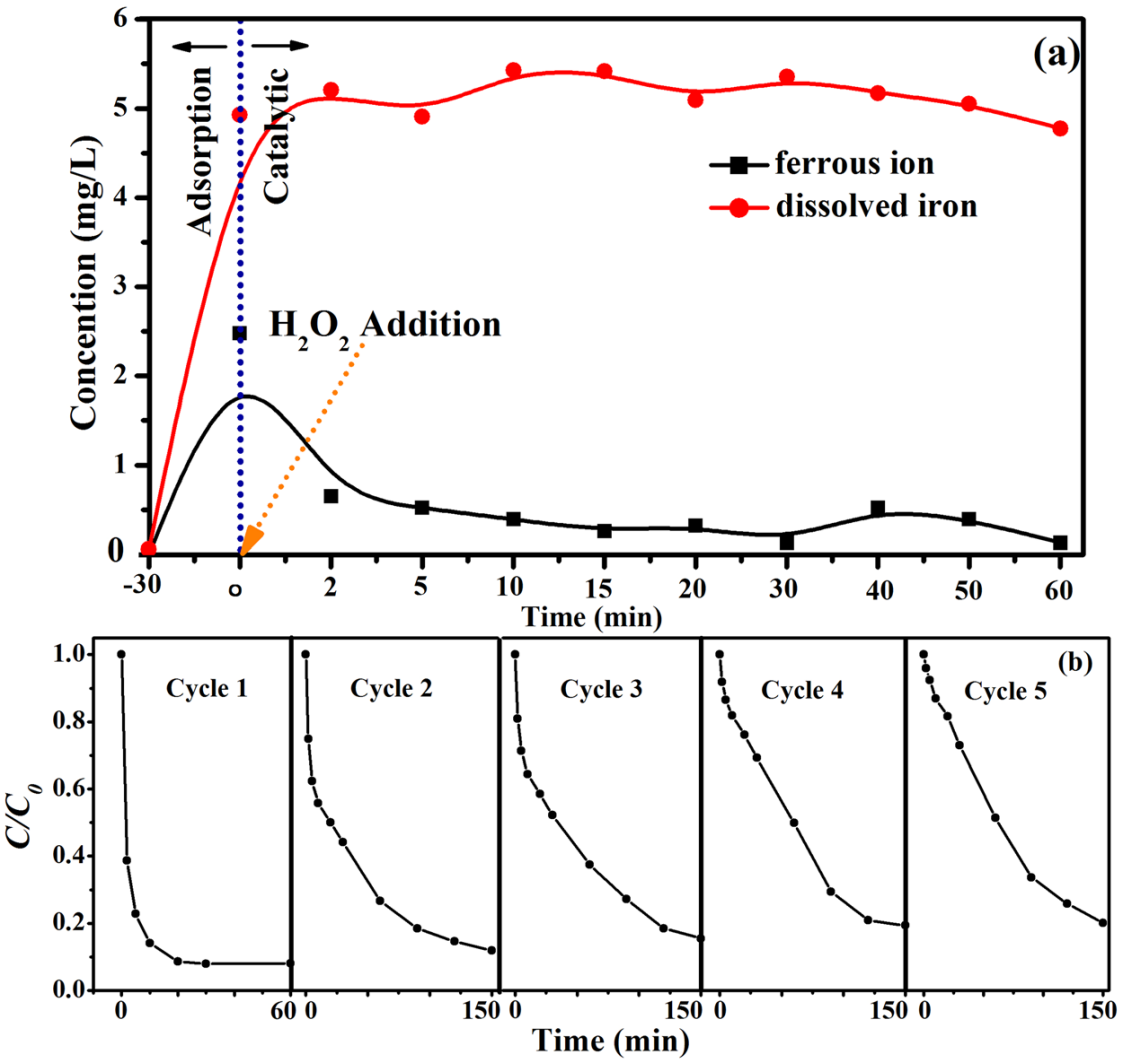
(**a**) Variation of the ferrous ion and dissolved iron in the solution during EtBr degradation under the standard reaction conditions. (**b**) Recycling properties of heterogeneous Fenton degradation of EtBr over A-ATP@Fe_3_O_4_ under the standard reaction conditions.

To investigate the effects of the concentrations of dissolved Fe on the degradation of EtBr, the heterogeneous Fenton reaction was performed under the standard reaction condition. As shown in Fig. 6(a), in the adsorption stage, the concentration of ferrous ion increased gradually and reached a peak value of 2.48 mg/L, at which about 60% of EtBr was absorbed. After H_2_O_2_ was added, the concentration of Fe^2+^ decreased to about 0.26 mg/L where the removal rate of EtBr was 90% after 20 min. The reason is that the catalyst can release ferrous ions to the acid solution, and H_2_O_2_ can oxidize the ferrous ions to generate ∙OH^50^. Different from other heterogeneous Fenton reaction, a small amount of ferrous ion was released from the catalyst in the catalytic degradation stage. However, the fast degradation rate at 2 min implies that H_2_O_2_ exhibited an excellent ability to oxidize ferrous ions to produce ∙OH quickly, leading to a fast decreasing of ferrous ions. Further, the variation of ferrous ions also caused the increase of the dissolved iron from the A-ATP@Fe_3_O_4_ composite and the oxidation of ferrous ions in solution. The dissolved iron amounted to 5 mg/L, which is equivalent to about 0.62% of total iron in the catalyst (1.5 g/L).

### Reusability of A-ATP@Fe_3_O_4_

The spent A-ATP@Fe_3_O_4_ was recycled and reused for EtBr degradation under standard reaction condition. As shown in Fig. 6(b), A-ATP@Fe_3_O_4_ maintained more than 80% of its catalytic capacity after five successive runs in 150 min of reaction. The reduced EtBr degradation efficiency probably resulted from the reduction of released iron ions from the catalyst in each successive runs. Therefore, to maintain an adequate quantity of the catalyst in the aqueous solutions and thus maintain the degradation efficiency, a prolonged degradation time may be needed.

### Possible degradation mechanisms

**Figure 7:**
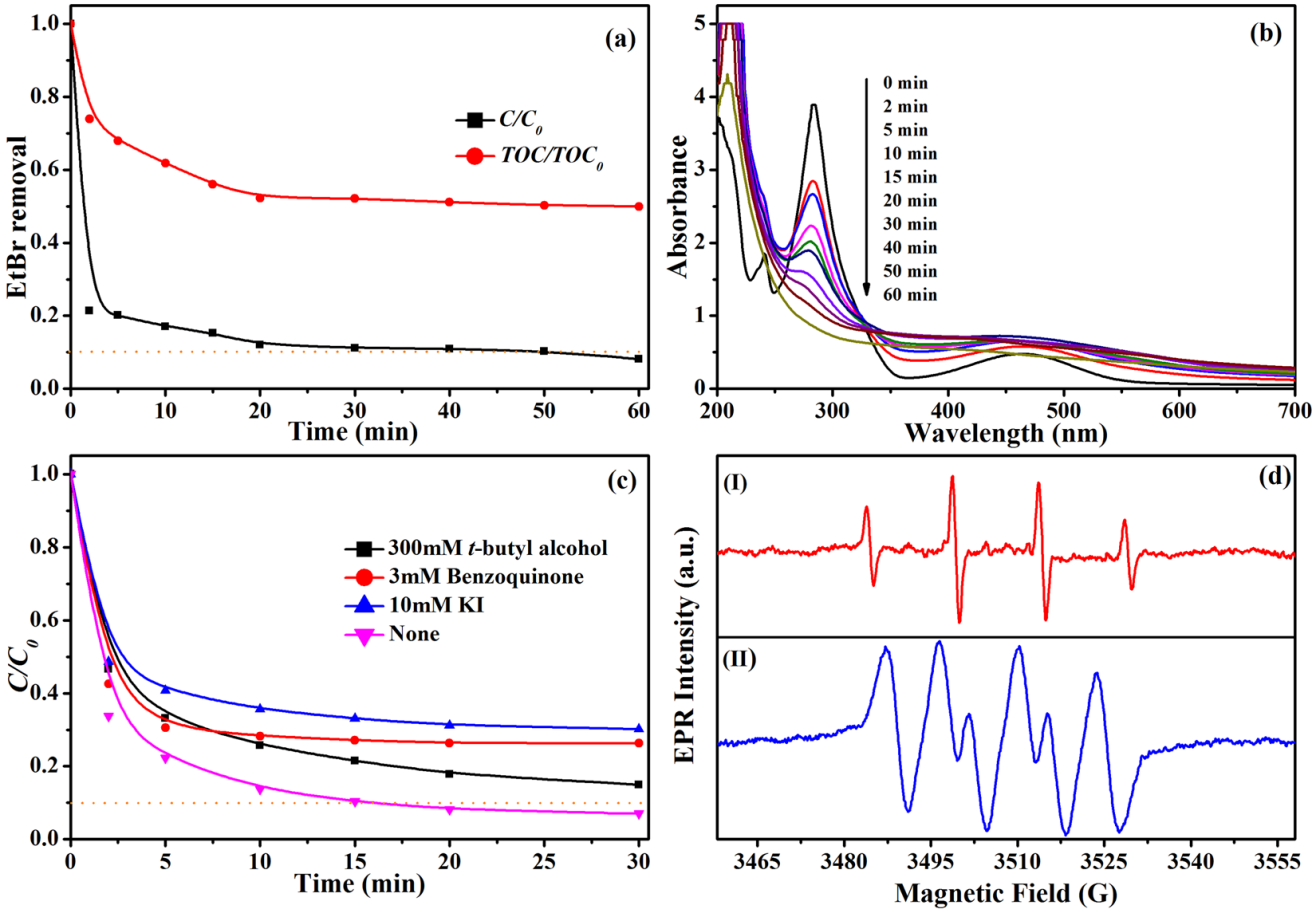
(**a**) Temporal change in EtBr and TOC removal. (**b**) UV-vis absorption spectra of the EtBr solution during the reaction in the systems of A-ATP@Fe_3_O_4_ (**c**) Effect of radical scavengers on the degradation of EtBr, and Reactions were conducted under the standard conditions. (**d**) The EPR spectra for the DMPO−∙OH (I) and DMPO−∙O_2_^−^ (II) of the A-ATP@Fe_3_O_4_ catalytic system with H_2_O_2_.

The EtBr degradation process was indicated by mineralization (reduction of TOC) (Fig. 7(a)) and the UV–visible absorption spectrum. The results indicated that the maximum TOC removal rate was approximately 45% after 20 min, suggesting that 45% of EtBr was oxidized by active species(∙OH, ∙O_2_^−^, etc.) to CO_2_ and H_2_O. This result is confirmed by the UV–vis absorption spectra of EtBr (Fig. 7(b)), which showed that the characteristic peak at *λ* = 285 nm became smaller and almost disappeared as the degradation proceeded. In addition, we observed that the absorption peak at *λ* = 241 nm weakened sharply after the addition of H_2_O_2_, indicating that the attack by the highly reactive hydroxyl radicals led to rapid opening of the benzene ring^51^. In addition, a new absorption peak at *λ* = 210 nm was recorded after 2 min of treatment and decreased gradually with reaction time. This may suggest that EtBr was attacked by hydroxyl radicals to produce a large number of intermediate products^52^, although further confirmation is needed.

To confirm reactive species in the degradation process, *t*-butyl alcohol and benzoquinone (BQ) were selected as radical scavengers during EtBr degradation, respectively. Excess *t*-butyl alcohol could scavenger all of the ∙OH produced by the system in the solution^53^. As shown in the Fig. 7(c), the degradation reaction rate of EtBr decreased evidently after the addition of *t*-butyl alcohol, which indicated the existence of ∙OH. However, about 20% of EtBr was not affected by the presence of *t*-butyl alcohol, which suggests the existence of other reactive species. After that, BQ was added to the system as a scavenger of ∙O_2_^−^^54^. From Fig. 7(c), with the addition of excess BQ, the EtBr degradation decreased from 68%, 77%, 86%, and 90% (in the absence of BQ) to 56%, 69%, 70%, and 72% at 2, 5, 10, and 15 min, respectively, suggesting that EtBr was also oxidized by the attack of ∙O_2_^−^ in the solution. Further, the electron paramagnetic resonance (EPR) technique was used to further confirm the direct involvement of ∙OH and ∙O_2_^−^ in the degradation process. As shown in Fig. 7(d,I), the characteristic 1:2:2:1 quartet signal was detected using 5,5-dimethyl-1-pyrroline *N*-oxide (DMPO) as a spin trapping agent, indicating the existence of ∙OH during the heterogeneous Fenton reaction. Besides, because ∙O_2_^−^ was extremely unstable in aqueous solution, methanol was used as a solvent for detection of ∙O_2_^−^. Figure 7(d,II) clearly shows that the six characteristic peaks of DMPO-∙O_2_^−^ existed in the degraded system.

**Figure 8:**
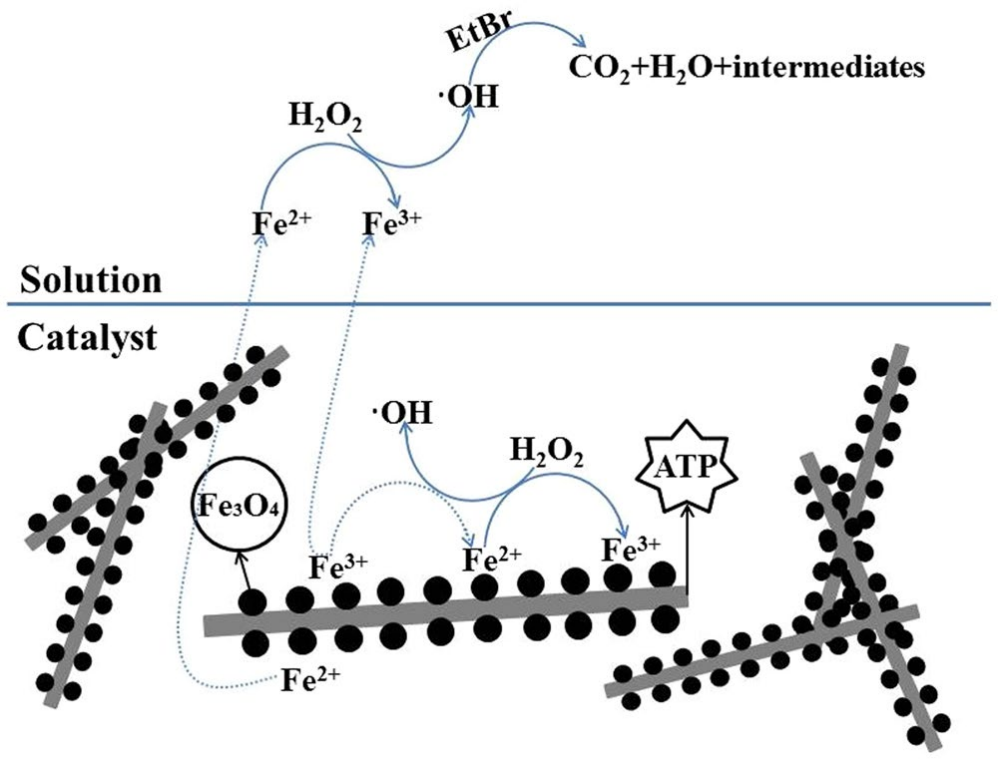
Schematic diagram of the reaction mechanism of the H_2_O_2_ activation by A-ATP@Fe_3_O_4_ catalyst under acidic condition.

Combining the results from the reactive oxygen species assay and the EPR analysis, we determined that ∙OH and ∙O_2_^−^ were present in the catalytic system. Thus, the possible reaction mechanism of H_2_O_2_ activation by A-ATP@Fe_3_O_4_ under acidic condition is illustrated in Fig. 8. According to our observations, it is possible that Fe^2+^ and Fe^3+^ from partial dissolution of iron oxides under acidic conditions initiate the decomposition of H_2_O_2_ through a homogeneous Fenton chain reaction. Initially, the dissolved Fe^2+^ can react with H_2_O_2_ to generate Fe^3+^ and ∙OH (equation (1)), which yield HO_2_∙/∙O_2_^−^ and simultaneously produce Fe^2+^ (equation (2)). The generated ∙OH (equation (1)) may further react with H_2_O_2_ to generate HO_2_∙/∙O_2_^−^ (equation (3)). Then partial Fe^2+^ is converted to Fe^3+^ through oxidation of the produced ∙OH (equation (4)). Thus, all of these reactions lead to cycling of iron ions in the solution system. In our work, a large number of H^+^ ions in solution facilitate that the reactions in equations (1) and (4), leading to decreased Fe^2+^ along with increased Fe^3+^ during the catalytic reactions.

Moreover, the amount of EtBr degradation declined from 68%, 77%, 86%, and 90% to 51%, 59%, 64%, and 66% at 2, 5, 10, and 15 min in the presence of excess KI (10 mM), suggesting that ∙OH originating from the surface of the catalyst played a dominant role in EtBr degradation (Fig. 7(c)). And the total amount of iron dissolved remained constant (Fig. 6(a)), implying that not much Fe^2+^ was released in EtBr degradation. Therefore, we conjecture that similar chain reactions occurred on the surface of the catalyst, which is different from the that in solution. Moreover, the hydroxyl radicals generated on the catalyst surface by the chain reaction are also involved in the degradation of EtBr. This may explain the reduction of catalytic performance in the process of reusing, which is similar with the result reported by Luo *et al*.^55^.

1 $${{\rm{Fe}}}^{2+}+{{\rm{H}}}_{2}{{\rm{O}}}_{2}+{{\rm{H}}}^{+}\to {{\rm{Fe}}}^{3+}+\cdot {\rm{OH}}+{{\rm{OH}}}^{-}$$

2 $${{\rm{Fe}}}^{3+}+{{\rm{H}}}_{2}{{\rm{O}}}_{2}\to {{\rm{Fe}}}^{2+}+{{\rm{HO}}}_{2}\cdot +{{\rm{H}}}^{+}$$

3 $${{\rm{H}}}_{2}{{\rm{O}}}_{2}+\cdot {\rm{OH}}\to \cdot {{\rm{O}}}_{2}^{-}+{{\rm{H}}}_{2}{\rm{O}}$$

4 $${{\rm{Fe}}}^{2+}+\cdot {\rm{OH}}\to {{\rm{Fe}}}^{3+}+{{\rm{OH}}}^{-}$$

5 $$\cdot {\rm{OH}}+\cdot {{\rm{O}}}_{2}^{-}({{\rm{HO}}}_{2}\cdot )\to {{\rm{O}}}_{2}+{{\rm{OH}}}^{-}(+{{\rm{H}}}_{2}{\rm{O}})$$

6 $$\cdot {\rm{OH}}+\cdot {\rm{OH}}\to {{\rm{H}}}_{2}{{\rm{O}}}_{2}$$

7 $$\cdot {\rm{OH}}+{\rm{EtBr}}\to {{\rm{CO}}}_{2}+{{\rm{H}}}_{2}{\rm{O}}+{\rm{intermediates}}$$

## Conclusion

In this study, a heterogeneous Fenton catalyst ATP@Fe_3_O_4_ was successfully synthesized by introducing nanoscaled Fe_3_O_4_ particles onto the treated ATP surface via co-precipitation. The obtained nanocomposites were carefully characterized which confirmed Fe_3_O_4_ nanoparticles with an average size of 15 nm was spread on the surface of ATP samples after different treatments. The as-prepared ATP@Fe_3_O_4_ nanocomposites all exhibited good sorptive removal ability for EtBr. Compared to P-ATP@Fe_3_O_4_, A-ATP@Fe_3_O_4_ has greater sorptive and catalytic capacity for EtBr. The higher sorptive capacity of A-ATP@Fe_3_O_4_ nanocomposites was attributed to larger specific surface area, while degradation of EtBr was mainly ascribed to more abundant hydroxyl radicals (∙OH). The highest degradation rate of EtBr and TOC removal were 92% and 45% within 60 min under optimal operation conditions: *T* = 323 K, 30 mmol/L H_2_O_2_, 1.5 g/L A-ATP@Fe_3_O_4_, and pH 2.0. Moreover, A-ATP@Fe_3_O_4_ composites have good regeneration ability and could be separated by external magnet. Thus, A-ATP@Fe_3_O_4_ nanocomposites can be safely used to remediate organic contaminants from aqueous solution.

## Materials and Methods

### Chemicals and materials

All the reagents used in the experiment were of analytical reagent grade and used without further purification. ATP was provided by Xuyu Clay Technology Co., Jiangsu China. Hydrogen peroxide (H_2_O_2_, 30% (w/w)), ethanol, *t*-butyl alcohol, potassium iodide (KI), hydrochloric acid (HCl), sodium hydroxide (NaOH), ferric chloride hexahydrate (FeCl_3_·6H_2_O), ferrous sulfate heptahydrate (FeSO_4_·7H_2_O), and high-purity compressed nitrogen (N_2_) gases were obtained from Yangzhou Chemicals Corporation (Yangzhou, China). EtBr and BQ were purchased from Shanghai Aladdin Bio-Chem Technology Co., Ltd (Shanghai, China).

### Preparation and characterization of catalyst

The pristine ATP powder was purified as follows. Briefly, 20 g of ATP were dispersed in 300 mL of deionized water and stirred for 2 h. The resulting slurry was then settled for 2 h, and the supernatant was decanted to remove impurities. Next, the prepared ATP was immersed in 300 mL of deionized water with 200 mL of H_2_O_2_ (30%, w/w). The suspension was magnetically stirred for 5 h, and sonicated for 30 min (40 kHz). The suspension was centrifuged at 5000 rpm, and the resulting precipitate was vacuum dried for 24 h at 70 °C and stored for subsequent use.

P-ATP was then activated in 150 mL of 1 M HCl with constant magnetic stirring for 5 h followed by sonication for 30 min. A-ATP was vacuum filtered, washed with deionized water and ethanol to a pH value of 6–7, and then dried for 24 h at 70 °C under vacuum.The prepared ATP was stored in a desiccator at room temperature for further use.

A-ATP@Fe_3_O_4_ were synthesized by co-precipitation method^56^. A-ATP (2.0 g) were added to 240 mL of deionized water in a 500 mL flask and stirred for 6 h. Then, the pH of the suspension was adjusted to 8 using 5 M NaOH solution. The stable suspension was bubbled with a constant N_2_ flow for 30 min to remove the dissolved oxygen^57^. Next, a 0.6 mol/L FeSO_4_ solution (20 m L) was added to the flask and sonicated for 30 min, followed by addition of 0.8 mol/L FeCl_3_ solution (20 ml). The mixture was then sonicated for 15 min. NaOH solution (5 M) was added to the flask drop wisely. Black precipitate appeared immediately after NaOH addition, and the reaction was terminated at a pH of 10. The nanocomposites were aged at 60 °C for another 1.5 h. The suspension was centrifuged at 4000 rpm for 5 min; the as-prepared Fe_3_O_4_ loaded ATP was washed with deionized water and ethanol several times to remove free ions and dried in a vacuum oven at 70 °C for 24 h. Finally, A-ATP@Fe_3_O_4_ nanoparticles with an Fe_3_O_4_-to-ATP mass ratio of 1:1 were obtained. In addition, P-ATP@Fe_3_O_4_ was prepared following the above procedure without HCl activation and Fe_3_O_4_ alone was synthesized without adding ATP. All the products were stored in a desiccator under room temperature before use.

### Characterization of catalyst

The morphology of the catalyst was observed on a scanning electron microscope (S-4800II, Japan) operated at an acceleration voltage of 15 kV. The surface groups of the nanocomposites were recorded by a micro infrared spectrometer (Cary 610/670, USA). The phase structure of the nanocomposites was obtained by XRD analysis (D8 Advance Bruker AXS, Germany). EDS were measured using a X-ray energy dispersive spectrometer (Thermo Electron Corporation) with Al Kα radiation as the excitation source. XPS (ESCALAB 250 Xi, USA) was used to identify the metal oxidation states of the nanocomposites. The magnetization of ATP-Fe_3_O_4_ and Fe_3_O_4_ was measured at room temperature using vibrating sample magnetometry (VSM-EV7, ADE) with a maximum applied field of 1.7 T. The specific surface area of the catalysts was determined by N_2_-BET analysis using an accelerated surface area and a porosimetry analyzer (ASAP 2460).

### Degradation experiment

Batch degradation experiments of EtBr were conducted in a conical flask (250 mL) incubated in a water bath with a constant temperature oscillator (TZ-2EH, Beijing Wode Co.) and shaken at 150 rpm in darkness. The reaction suspension was prepared by adding the required amount of catalyst (0.2–2.0 g/L) to 200 mL of an 80 mg/L EtBr solution at different pH values (2.0–9.0). The suspension was vibrated for 30 min to achieve the adsorption/desorption equilibrium. The EtBr concentration after equilibrium was measured and considered as the initial concentration (*C*_*0*_). Then, a known concentration of H_2_O_2_ was added to initiate the degradation reaction. Subsamples were taken at set intervals during the reaction using a 3 mL centrifuge tube and immediately centrifuged at 5000 rpm for 5 min using an H1650-W centrifuge (HuNan) to remove the catalyst. The EtBr concentration of the supernatant was determined at *λ* = 285 nm by using a UV–visible spectrophotometer. Each experiment was run in triplicate.

To test the regeneration ability of Fe_3_O_4_-ATP, spent nanoparticles were separated from the suspension when the EtBr was almost completely degraded. The regenerated sorbents were then used again for EtBr degradation. The regeneration process was repeated five times.

### Analytical methods

Total organic carbon (TOC) was analyzed using a TOC-LCPN analyzer (Shimadzu, Japan). The component and content of different elements were measured by an energy dispersive X-ray Spectrometer. The concentration of EtBr was analyzed using an intelligent UV-2501PC/2550 detector (ShangHai) at *λ* = 285 nm.

The presence of hydroxyl radicals and superoxide radical were determined using *t*-butyl alcohol, KI and BQ, respectively, as scavengers. The effective radicals that appeared in the degradation process were further detected by electron spin resonance (A300–10/12, Bruker, Germany).The concentration of ferrous ions was measured colorimetrically with 1,10-phenanthroline at *λ* = 510 nm on a UV–vis spectrophotometer^58^. The total dissolved iron was analyzed by atomic absorption spectroscopy (G8433A).

## Electronic supplementary material

Supplementary Information
