# Supplementary material for: Magnetic Activated-ATP@Fe3O4 Nanocomposite as an Efficient Fenton-Like Heterogeneous Catalyst for Degradation of Ethidium Bromide
Source: Sci Rep. 2017 Jul 20;7:6070. doi: 10.1038/s41598-017-06398-3 (PMC5519544; doi:10.1038/s41598-017-06398-3)
Supplement: Supplementary file 1 — Supplementary Information [file 41598_2017_6398_MOESM1_ESM.doc]

**Magnetic Activated-ATP@Fe_3_O_4_ Nanocomposite as an Efficient Fenton-Like Heterogeneous Catalyst for Degradation of Ethidium Bromide**

Shuwen Han^1^, Hemin Yu^1^, Tingting Yang^1^, Shengsen Wang^1,2^, Xiaozhi Wang ^1,2^ *

^1^College of Environmental Science and Engineering, Yangzhou University, Jiangsu 225127, China.

^2^Jiangsu Collaborative Innovation Center for Solid Organic Waste Resource Utilization, Nanjing 210095, China.

*Corresponding Author: Xiaozhi Wang, Email: xzwang@yzu.edu.cn.

Tel.: +86 514 87991081; Fax: +86 514 87979528.

**Supplementary Figure S1.** Nitrogen adsorption-desorption isotherm (a), and BJH corresponding pore size distribution curve (b) of P-ATP@Fe_3_O_4_, A-ATP@Fe_3_O_4_, and Fe_3_O_4_.

**Supplementary Figure S2.** FT-IR spectra of P-ATP@Fe_3_O_4_ and A-ATP@Fe_3_O_4_ nanocomposites. The original P-ATP and A-ATP serves as control.
